# Cooperative stimulation of vascular endothelial growth factor expression by hypoxia and reactive oxygen species: the effect of targeting vascular endothelial growth factor and oxidative stress in an orthotopic xenograft model of bladder carcinoma

**DOI:** 10.1038/sj.bjc.6602522

**Published:** 2005-04-19

**Authors:** N S Brown, E H Streeter, A Jones, A L Harris, R Bicknell

**Affiliations:** 1 Molecular Angiogenesis Group, Cancer Research UK, Weatherall Institute of Molecular Medicine, University of Oxford, John Radcliffe Hospital, Oxford OX3 9DS, UK; 2 Molecular Oncology Group, Cancer Research UK, Weatherall Institute of Molecular Medicine, University of Oxford, John Radcliffe Hospital, Oxford OX3 9DS, UK; 3 Department of Urology, Churchill Hospital, Oxford OX3 7LJ, UK

**Keywords:** angiogenesis, hypoxia, oxidative stress, thymidine phosphorylase, VEGF

## Abstract

Elevated thymidine phosphorylase has been shown to correlate with increased angiogenesis and poor prognosis in many cancers including transitional cell carcinoma of the bladder. *In vitro* studies have demonstrated that thymidine phosphorylase activity causes cellular oxidative stress and increases secretion of vascular endothelial growth factor. In this study, we show that thymidine phosphorylase activity also augments levels of the hypoxia-inducible factor-1*α* during *in vitro* hypoxia, and that thymidine phosphorylase activity and hypoxia act in concert to increase vascular endothelial growth factor (VEGF) secretion. We also demonstrate that thymidine phosphorylase overexpression confers tumorigenicity on an orthotopically implanted transitional cell carcinoma cell line. Administration of the antioxidant *N*-acetylcysteine together with a blocking anti-VEGF antibody abrogates the increase in tumorigenicity. Our results support the increased efficacy of combination approaches to antiangiogenic therapy.

While bladder cancer was responsible for around 46 000 deaths in Europe and North America in 2000, there were 151 000 new diagnoses that year. This difference between incidence and death rate is due to the heterogeneous clinical behaviour of the transitional cell carcinomas (TCCs) that account for almost all bladder tumours in the developed world. 70–80% of TCCs present as superficial, papillary tumours (stages Ta and T1). With treatment, 5-year survival in these patients should approach 90–95%, with the majority of patients either cured, or dying with (not from) their bladder cancer. Unfortunately, for the 20–30% of tumours that present at stages T2–T4, and the minority of superficial tumours that progress despite treatment, 5-year survival rates are much lower. The prognosis of patients with muscle-invasive carcinoma is poor for two reasons. Firstly, invasion promotes local, lymphatic and haematogeneous dissemination. In addition, invasive disease necessitates radical surgery or radiotherapy, with the morbidity these entail. There is therefore a need to identify the mechanisms that increase tumorigenicity in TCC.

The ‘angiogenic enzyme’ thymidine phosphorylase (TP) is commonly upregulated in many solid tumours including TCC (O'Brien *et al*, 1996), and overexpression has been shown to correlate with increased tumour invasion (O'Brien *et al*, 1996; Sawase *et al*, 1998) and poor prognosis (Arima *et al*, 2000) in bladder carcinoma. While TP has been known to be angiogenic for many years (Ishikawa *et al*, 1989), the precise mechanism has remained elusive. We have previously shown that TP activity induces oxidative stress in a TCC cell line, possibly via the production of strongly reducing sugars that generate oxygen free radicals during the early steps of protein glycation (Brown *et al*, 2000). One consequence of TP activity in this cell line is increased expression of the angiogenic factor vascular endothelial growth factor (VEGF) (Brown *et al*, 2000). Increased VEGF expression has been shown to correlate with recurrence and progression in bladder tumours (Crew *et al*, 1997). Transcription of the VEGF gene is known to be driven by the hypoxia-inducible factor HIF-1*α*, and recent evidence has suggested that cellular HIF-1*α* levels are raised not only by hypoxia but are also increased in the presence of reactive oxygen species (ROS) (Page *et al*, 2002; Gao *et al*, 2002). We have therefore conducted *in vitro* experiments to determine whether hypoxia and TP activity can cooperatively increase VEGF production by a TCC cell line.

As noted above, elevated TP has been shown to correlate with aggressive TCC behaviour in clinical studies. It is not, however, possible to use observational clinical data to prove that TP activity is causal. Thymidine phosphorylase levels might be increased secondary to the microenvironmental changes that accompany tumour growth and invasion. For instance, cellular TP levels are known to be highly responsive to the local cytokine milieu (Goto *et al*, 2001). To demonstrate causality, we have compared the *in vivo* behaviour of two TCC cell lines, one transfected with the TP gene (RT112-TP), the other an empty vector control (RT112-EV). Differences in the tumorigenicity of TCC cell lines are best demonstrated by orthotopic implantation, rather than xenografting into the mouse flank (Kerbel *et al*, 1991). We have therefore implanted RT112-TP and RT112-EV into the bladder walls of athymic rats, via laparotomy. Finally, we aimed to determine whether ROS and VEGF might increase the tumorigenicity of TP-overexpressing TCC *in vivo*. If this is the case, then therapies that diminish tumour cell oxidative stress and counter VEGF activity should reduce the tumorigenicity of TP-overexpressing carcinomas. To test this theory *in vivo*, we have treated rats with the antioxidant *N*-acetyl cysteine (NAC) and an anti-VEGF monoclonal antibody, to determine the effect of these interventions upon the tumorigenicity of RT112-TP.

## MATERIALS AND METHODS

### Materials

The cell lines RT112-EV and RT112-TP have previously been described (Brown *et al*, 2000). VG76e is an anti-human VEGF mouse monoclonal antibody that has previously been shown to block VEGF activity, both by its inhibition of VEGF-dependent growth stimulation of human umbilical vein endothelial cells *in vitro*, and its abrogation of angiogenesis in the primate corpus luteum *in vivo* (Fraser *et al*, 2000).

### Cell culture for *in vitro* experiments

RT112-EV and RT112-TP cells were cultured in DMEM containing 25 mM glucose and 10% fetal calf serum. At 16 h prior to the start of the experiment, cells were transferred to DMEM containing 5.5 mM glucose, 0.01% bovine serum albumin, 45 *μ*g ml^−1^ streptomycin, 45 *μ*g ml^−1^ penicillin, and 40 *μ*g ml^−1^ kanamycin (Life Technologies, Paisley, UK).

### Immunoblotting

RT112-EV and RT112-TP cells were cultured to around 80% confluence. Cells were then fed with control medium, or medium containing 500 *μ*M thymidine, for 10 h. After this, the cells were placed in normoxia or hypoxia (0.1% oxygen) for a further 6 h. At harvest, the cells were lysed in sodium phosphate/SDS buffer (pH 7.2) containing ‘Complete, EDTA-free’ protease inhibitors (Roche Diagnostics, Lewes, UK). Lysates were centrifuged to remove particulates, and the protein concentration determined using the bicinchonic acid protein assay (Pierce & Warriner, Chester, UK). Protein lysate (50 *μ*g) was separated by electrophoresis on a 12% acrylamide gel and transferred to an Immobilon-P membrane (Millipore, Watford, UK). After blocking, the membrane was probed overnight at 4°C with anti-HIF-1*α* mouse monoclonal antibody NB100-123 diluted 1 : 750 (Novus Biologicals, Littleton, USA). Goat anti-mouse horseradish peroxidase-conjugated secondary antibody (Dako, Ely, UK) was used at 500 *μ*g l^−1^, and HIF-1*α* visualised using the Renaissance enhanced luminol system (New England Nuclear, Hounslow, UK) and Hyperfilm (Amersham Pharmacia Biotech, Little Chalfont, UK). The membrane was then stripped by two 5-min rinses in 200 mM sodium hydroxide, followed by two 5-min rinses in distilled water, reblocked and immunoblotted for haem oxygenase-1 (HO-1), using the anti HO-1 mouse monoclonal antibody OSA-110 at 0.6 *μ*g ml^−1^ (Stressgen, Victoria, Canada). After visualisation of the HO-1 bands, the membrane was again stripped, and probed with the anti *β*-tubulin mouse monoclonal antibody T4026 at 0.26 *μ*g ml^−1^ (Sigma-Aldrich, Poole, UK).

### ELISA experiments

RT112-TP cells were seeded into six-well plates at 1 × 10^5^ cells well^−1^ and cultured for 24 h. Triplicate data were obtained by allocating three wells of RT112-TP to each treatment. The growth medium was replaced with serum-free medium containing 5.5 mM glucose, and the cells incubated for an additional 24 h. The cells were then treated with 0 or 200 *μ*M thymidine for 6 h, and then transferred to 21 or 0.1% oxygen for the remaining 10 h of the experiment. Some wells were treated with 50 *μ*M of the TP inhibitor 5-chloro-6-[1-(2-iminopyrrolidinyl)methyl] uracil hydrochloride (TPI), a gift from Dr M Fukushima, Taiho Pharmaceutical Co. Ltd, Saitama, Japan. Conditioned medium was collected, centrifuged to remove particulates, and frozen at −80°C for future analysis by ELISA. Concurrently, the cells in each well were released by exposure to trypsin, and their number determined by Coulter Counter. The VEGF concentration in the conditioned medium was then determined by ELISA (R&D Systems, Abingdon, UK). These results were then divided by the number of cells in the corresponding well, to obtain a value in picograms of VEGF produced per million cells per 16 h. The data were examined for significance by Kruskall–Wallis analysis.

### Orthotopic implantation of RT112-EV and RT112-TP cells into the immunodeficient rat bladder

Nude 3-week-old immunodeficient rats were purchased from Harlan UK Ltd, (Bicester, UK). The cell lines RT112-EV and RT112-TP were prepared by retroviral transfection of the RT112 cell line (Brown *et al*, 2000). Orthotopic implantation of RT112-TP and RT112-EV into the nude rat bladder wall was performed essentially as described by Oshinsky *et al* (1995). Briefly, following anaesthesia the bladder was exposed via a 5 mm midline abdominal incision. In all animals, 5 × 10^6^ cells in a 100 *μ*l volume were injected via a 27 gauge needle directly into the bladder wall and the abdomen washed and sutured. Treatment schedules involved administration of NAC (0.1 g ml^−1^) *ad libitum* in the drinking water and/or a VG76e (500 *μ*g) bolus intraperitoneally (i.p.) twice weekly. Rats were killed 6 weeks after implantation and the bladders examined. Animal procedures were performed in accordance with the UK Home Office licence number 70/4949. Each experiment employed the minimum number of rats needed to obtain statistically meaningful results. Tumour take data were analysed by Fisher's exact test.

### Immunohistochemistry

Immunohistochemistry was performed using the standard ABC-HRP protocol. Paraffin-embedded sections were dewaxed, rehydrated and then microwaved twice in 10 mM sodium citrate buffer (pH 6.0). Endogenous peroxidase activity was blocked with 3% hydrogen peroxide (5 min at room temperature). After blocking with 1 : 20 fetal calf serum in PBS, the sections were incubated with primary antibody. Anti-HO-1 (Calbiochem, Nottingham, UK) and anti-4-hydroxy-2-nonenal (Autogen Bioclear, Calne, UK) antisera were both rabbit anti-mouse. The Vector ABC-Elite kit (Vector Laboratories, Peterborough, UK) was used, and peroxidase activity was visualised with metal-enhanced diaminobenzidine (Perbio, Cramlington, UK).

## RESULTS

### Thymidine phosphorylase activity augments the hypoxic induction of HIF-1*α* in RT112-TP

**Figure 1:**
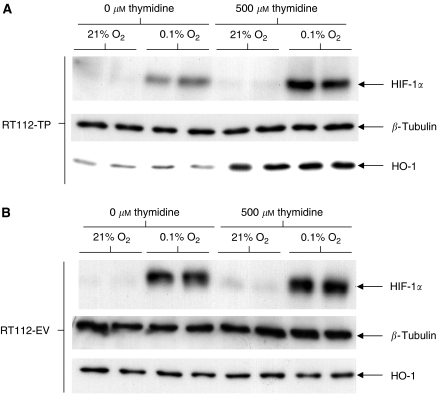
Treatment of RT112-TP with thymidine further augments HIF-1*α* levels under hypoxia. Duplicate dishes of RT112-TP and RT112-EV were exposed to thymidine and/or hypoxia. Immunoblotting for the hypoxia-inducible factor HIF-1*α* was then undertaken. In addition, levels of the marker of oxidative stress HO-1, and the loading control *β*-tubulin, were also assessed. As with previous *in vitro* studies (Brown *et al*, 2000), HO-1 levels were increased in RT112-TP treated with thymidine, but were not raised in thymidine-treated RT112-EV, suggesting that TP activity causes oxidative stress. The hypoxic induction of HIF-1*α* was greater in the plates where RT112-TP had been exposed to thymidine, implying a cooperative induction of HIF-1*α* by hypoxia and TP activity. In contrast, thymidine treatment of RT112-EV has no effect upon the hypoxic induction of HIF-1*α* in this control cell line.

RT112-EV and RT112-TP cells were exposed to thymidine and hypoxia, either alone or in combination, and levels of HIF-1*α* protein were analysed by immunoblotting. In addition, the levels of HO-1, a marker of oxidative stress, and the loading control *β*-tubulin were also measured. This was performed by repeat immunoblotting of the same membrane (Figure 1). As predicted by previous *in vitro* studies (Brown *et al*, 2000), HO-1 levels were increased in RT112-TP treated with thymidine, suggesting that these cells are under oxidative stress due to TP activity. An additional, novel, finding was that coexposure of the RT112-TP cells to thymidine and hypoxia gave greater induction of HIF-1*α* protein than did exposure to hypoxia alone. Exposure to thymidine alone did not increase levels of HIF-1*α*. Treatment of RT112-EV with thymidine did not induce HO-1, and the hypoxic induction of HIF-1*α* was not augmented by thymidine treatment of RT112-EV.

### Hypoxia and thymidine additively increase the secretion of VEGF by RT112-TP

**Figure 2:**
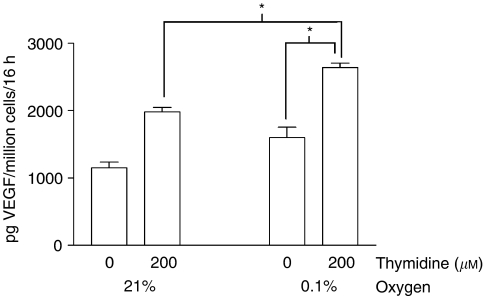
RT112-TP treated with thymidine and hypoxia secrete significantly more VEGF than cells receiving either treatment alone. Triplicate wells of RT112-TP were exposed to one of four treatments (0 *μ*M thymidine+21% oxygen; 200 *μ*M thymidine+21% oxygen; 0 *μ*M thymidine+0.1% oxygen; 200 *μ*M thymidine+0.1% oxygen). Mean VEGF production (+one standard error), expressed as picogram VEGF/million cells/16 h of culture is shown. As expected, both thymidine and hypoxia alone increased VEGF secretion. However, RT112-TP treated with both thymidine and hypoxia produced significantly more VEGF than those treated with thymidine alone (*P*=0.0495, Kruskall–Wallis analysis), and also secreted significantly more VEGF than RT112-TP exposed to hypoxia alone (*P*=0.0495, Kruskall–Wallis analysis). (^*^=significant difference by Kruskall–Wallis analysis).

**Figure 3:**
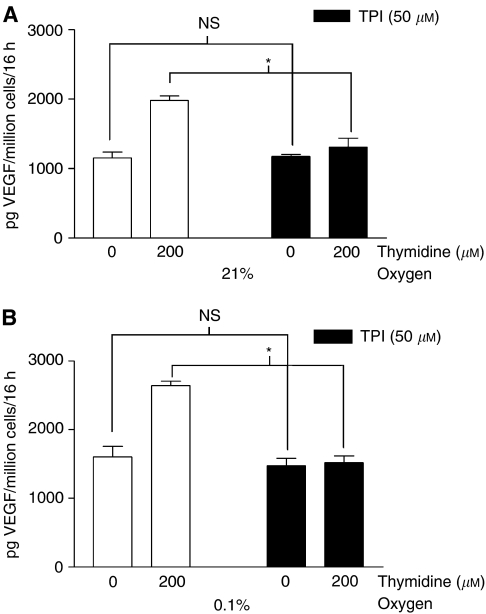
Increased VEGF production by thymidine-treated RT112-TP is due to TP activity in both normoxic and hypoxic conditions. During the same experiment as shown in Figure 2, triplicate wells of RT112-TP were also exposed to thymidine in the presence of 50 *μ*M TPI, a specific inhibitor of TP activity (Matsushita *et al*, 1999), under normoxic (**A**) and hypoxic (**B**) conditions. Mean VEGF production (+one standard error) is expressed as picogram VEGF/million cells/16 h of the culture. The data for the TPI-free wells are taken from Figure 2. (**A**) In normoxia, TPI abolished the thymidine-dependent increase in VEGF secretion by RT112-TP (*P*=0.0495, Kruskall–Wallis analysis), but had no effect upon baseline VEGF production (*P*=0.51, Kruskall–Wallis analysis). (**B**) In hypoxia, TPI abolished the thymidine-dependent increase in VEGF secretion by RT112-TP (*P*=0.0495, Kruskall–Wallis analysis), but had no effect upon baseline VEGF production (*P*=0.51, Kruskall–Wallis analysis). These results confirm that TP activity is responsible for the increased VEGF production observed in thymidine-treated RT112-TP. (NS=no significant difference by Kruskall–Wallis analysis. ^*^=significant difference by Kruskall–Wallis analysis).

Figure 2 shows VEGF production by the cell line RT112-TP *in vitro*, following exposure to hypoxia and/or thymidine. Both hypoxia alone and thymidine alone induce VEGF. A combination of hypoxia and thymidine gave significantly higher VEGF levels than treatment with either agent alone. We have also demonstrated that TP activity is the cause of increased VEGF production in thymidine-treated RT112-TP, both in normoxic and hypoxic conditions. 50 *μ*M of ‘TPI’, a specific inhibitor of TP activity (Matsushita *et al*, 1999), significantly attenuated the increase in VEGF secretion seen with thymidine treatment of RT112-TP. TPI had no effect upon baseline levels of VEGF production, however. These findings applied to RT112-TP in normoxia (Figure 3A), and also to RT112-TP in hypoxic conditions (Figure 3B).

### Thymidine phosphorylase overexpressing RT112 human bladder carcinoma cells are more tumorigenic than control cells when implanted into the bladders of athymic rats

The two transfectant cell lines derived from the human TCC cell line RT112 were directly implanted into the bladder walls of athymic rats. Implantation of the empty vector control cell line RT112-EV led to the establishment of only one tumour in 14 implants. The TP-transfectant cell line RT112-TP, however, gave tumours in six out of 12 rats. Fisher's exact test was used to confirm that RT112-TP is significantly more tumorigenic than RT112-EV in this orthotopic xenograft model (*P*=0.03).

### Combination therapy with an antioxidant and an anti-VEGF antibody decreases the *in vivo* tumorigenicity of RT112-TP

**Figure 4:**
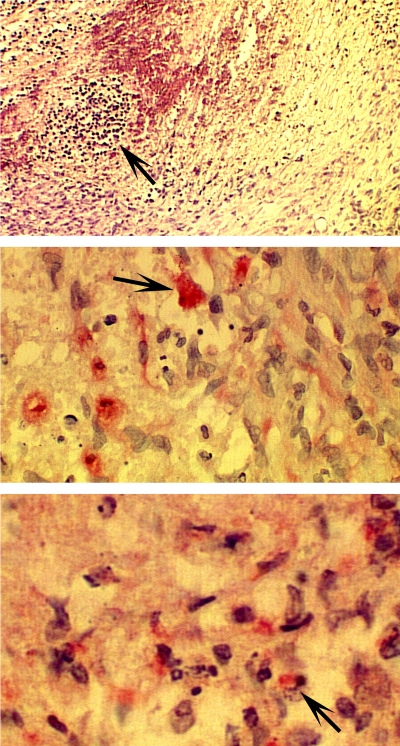
Immunohistochemical staining of the oxidative stress markers haem oxygenase-1 (HO-1) and 4-hydroxy-2-nonenal in RT112-TP tumours. The top panel is a low-power view with haematoxylin stain. The arrows indicate the sections stained for HO-1 (middle panel) and 4-hydroxy-2-nonenal (lower panel). The *ex vivo* RT112-TP tumour stained positive for both HO-1 and 4-hydroxy-2-nonenal. All normal tissues examined, including bladder, were negative for these markers (see Results).

It has previously been shown that TP activity correlates with cellular oxidative stress *in vitro* (Brown *et al*, 2000). To confirm the presence of oxidative stress in orthotopically implanted RT112-TP, we excised a RT112-TP tumour from the rat bladder and immunostained it for two known markers of oxidative stress. 4-Hydroxy-2-nonenal is the major end product of oxidative fatty acid metabolism and is generated by a free radical chain reaction mechanism during oxidative stress (Esterbauer *et al*, 1991), and 4-hydroxy-2-nonenal-modified proteins are markers of oxidative stress (Temma *et al*, 2004). Oxidative stress also induces haem oxygenase, and this can be used as a marker for stressed cells (Smith *et al*, 1994). The *ex vivo* tumour stained positive for both markers (Figure 4). Immunostaining was negative on the normal bladder, lung, liver and kidney. It has also been demonstrated that TP activity increases *in vitro* production of VEGF (Brown *et al*, 2000). Oxidative stress and VEGF activity may therefore play key roles in the increased *in vivo* tumorigenicity of TP-overexpressing carcinomas, and represent potential therapeutic targets. To examine their relative contribution to tumour take, we administered the antioxidant NAC and/or the anti-VEGF monoclonal antibody VG76e to 30 further rats carrying orthotopic implants of the TP overexpressing TCC cell line RT112-TP. Eight received no treatment, seven were given NAC (0.1 g ml^−1^ *ad libitum* in drinking water), eight were administered VG76e (500 *μ*g i.p. bolus dose twice weekly), and seven were subject to both interventions. After 6 weeks, four out of eight of the control group had developed a bladder tumour, but only two out of seven of the NAC group and two out of eight of the VG76e group showed evidence of tumour take. Of the seven rats receiving both treatments, none developed a tumour. The decreased tumour take in the rats receiving a single therapy was not statistically significant. The absence of tumours in the group given dual therapy was of borderline significance when eight rats from the second experiment alone were included in the control group (*P*=0.08, Fisher's exact test). It is valid, however, to expand the control group analysis to include rats from the first experiment, giving a control group with 10 out of 20 rats developing tumours. This is because the experiments were carried out under identical conditions, and the two control groups gave identical results. Analysis of this data using Fisher's exact test confirms that the tumorigenicity of RT112-TP *in vivo* is significantly decreased by coadministration of NAC and VG76e (*P*=0.03).

## DISCUSSION

The initial part of this study furthers our understanding of the mechanisms that link TP activity and tumour behaviour. We show that in the presence of hypoxia, TP activity further increases the induction of cellular HIF-1*α*. In contrast, others have shown that the enzymatic product of thymidine phosphorylase's action on thymidine, 2-deoxy-D-ribose, attenuates the hypoxic induction of HIF-1*α* in the leukaemic cell line HL-60 (Ikeda *et al*, 2002). It is therefore possible that different tumour cell lines may demonstrate considerable variation when studying the effects of TP activity upon HIF-1*α* induction. It has been known for several years that 2-deoxy-D-ribose reduces hypoxia-induced apoptosis in the cell lines KB-3-1 and HL-60 (Kitazono *et al*, 1998) (Ikeda *et al*, 2002). It would therefore be of future interest to determine whether TP activity promotes or represses hypoxia-induced apoptosis in our bladder carcinoma cell line. Heterogeneity of response when comparing cell lines may be further increased by the fact that HIF-1*α* is proapoptotic in some cell lines, but antiapoptotic in others (Greijer and van der Waal, 2004).

We have also shown that in the TP-transfectant bladder carcinoma cell line RT112-TP expression of the angiogenic factor VEGF was enhanced by the presence of both thymidine and hypoxia *in vitro*. These results confirm those of our previous study, and are of interest in the light of the findings of Nakajima *et al* (2004), who demonstrated that 2-deoxy-L-ribose, the stereoisomer of TP's product, suppressed the hypoxic expression of VEGF by the carcinoma cell line KB-3-1. Our results suggest that hypoxic stress, and oxidative stress caused by TP activity, may cooperatively stabilise HIF-1*α* and increase VEGF production in our TCC cell line. Initially, it appears counter-intuitive that a cell could experience both hypoxic and oxidative stress at the same time. Such concurrence is possible, however, because ROS exert their biological effects at extremely low concentrations – levels even lower than the concentration of molecular oxygen in a hypoxic environment.

We then demonstrated that TP overexpression significantly increases the tumorigenicity of bladder carcinoma cells implanted into the bladders of athymic rats. A large number of clinical research papers have shown a statistically significant correlation between TP expression and either tumour invasion or poor prognosis, but these observational studies cannot prove the presence of a causal relationship. In addition, the thymidine phosphorylase inhibitor (TPI) has been shown to reduce both tumour invasion and distant metastasis of a TP-expressing solid tumour in a mouse model (Takao *et al*, 2000). Jones and colleagues have previously used the *ex vivo* de-epithelialised rat bladder model to investigate the effects of TP upon the tumorigenicity of the RT112 cell line. They found that RT112-TP cells invaded the stroma of the *ex vivo* de-epithelialised rat bladder, while RT112-EV did not (Jones *et al*, 2002). Our experiments have shown for the first time that a TP-transfected bladder carcinoma cell line is significantly more tumorigenic than its empty vector control in the bladders of living rats. We believe that this strongly suggests a causal relationship between TP activity and aggressive tumour behaviour in the bladder.

Finally, we demonstrated that by administering both a monoclonal anti-VEGF antibody and the antioxidant NAC to the experimental animals, we could significantly reduce the tumorigenicity of RT112-TP in the nude rat bladder. We chose to investigate the therapeutic potential of an anti-VEGF monoclonal antibody because of evidence that VEGF may be an important mediator acting downstream of TP activity (Brown *et al*, 2000). *N*-acetyl cysteine was employed as our second agent because of its successful use in three previous studies. Firstly, the *in vitro* induction of carcinoma cell HO-1 by TP activity is abolished by NAC (Brown *et al*, 2000). The second study used the *ex vivo* de-epithelialised rat bladder model, and demonstrated that NAC reduced invasion of the stroma by a range of TCC cell lines (Fujiyama *et al*, 2001). Finally, Albini and colleagues have shown that NAC reduces tumorigenicity in a variety of animal models. For example, they have demonstrated that oral NAC inhibits the growth of a subcutaneously implanted Kaposi's sarcoma cell line in a murine model (Albini *et al*, 2001). Interestingly, they found VEGF production to be significantly reduced in the Kaposi's sarcomas from NAC-treated mice.

Anti-VEGF monoclonal antibodies and NAC are already in clinical use. The anti-VEGF antibody bevacizumab (Avastin) has recently completed large-scale phase III trials in advanced colorectal carcinoma (Kabbinavar *et al*, 2003) (Hurwitz *et al*, 2004). NAC (under its trade name ‘Parvolex’) has been the established first-line treatment of paracetamol overdose for many years. Both therefore have well-characterised clinical safety profiles, and may thus merit further investigation as therapeutic adjuncts in combination treatment of bladder carcinoma.
